# (6-Meth­oxy-2-oxo-2*H*-chromen-4-yl)methyl pyrrolidine-1-carbodithio­ate

**DOI:** 10.1107/S1600536812017953

**Published:** 2012-04-28

**Authors:** N. M. Mahabaleshwaraiah, K. Mahesh Kumar, O. Kotresh, Waleed Fadl Ali Al-eryani, H. C. Devarajegowda

**Affiliations:** a Department of Chemistry, Karnatak Science College, Dharwad 580 001, Karnataka, India; b Department of Physics, Yuvaraja’s College (Constituent College), University of Mysore, Mysore 570 005, Karnataka, India

## Abstract

In the title compound, C_16_H_17_NO_3_S_2_, the 2*H*-chromene ring is close to being planar [maximum deviation = 0.034 (2) Å] and the pyrrolidine ring is twisted about the C—C bond opposite the N atom. The dihedral angle between the ring-system planes is 75.24 (16)° and an intra­molecular C—H⋯S inter­action occurs. In the crystal, mol­ecules are linked by C—H⋯O hydrogen bonds and the packing also exhibits π–π inter­actions, with a distance of 3.6106 (13) Å between the centroids of the benzene rings of neighbouring mol­ecules.

## Related literature

For a related structure and background to the properties of coumarins, see: Kant *et al.* (2012 ▶). For further synthetic details, see: Shastri *et al.* (2004 ▶); Vasilliev *et al.* (2000 ▶).
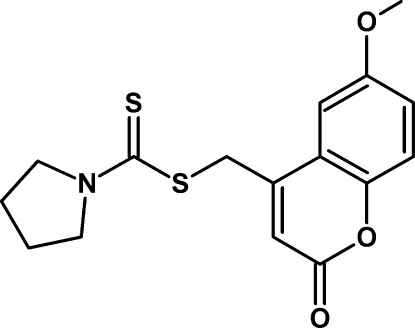


## Experimental

### 

#### Crystal data

C_16_H_17_NO_3_S_2_
*M* *_r_* = 335.43
Triclinic, 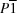

*a* = 6.7223 (2) Å
*b* = 8.0369 (2) Å
*c* = 15.4101 (5) Å
α = 75.320 (2)°
β = 88.482 (1)°
γ = 78.842 (1)°
*V* = 789.93 (4) Å^3^
*Z* = 2
Mo *K*α radiation
μ = 0.35 mm^−1^
*T* = 293 K
0.24 × 0.20 × 0.12 mm

#### Data collection

Bruker SMART CCD diffractometer
Absorption correction: multi-scan (*SADABS*; Bruker, 2001 ▶) *T*_min_ = 0.770, *T*_max_ = 1.000
15231 measured reflections
2768 independent reflections
2453 reflections with *I* > 2σ(*I*)
*R*_int_ = 0.024

#### Refinement

*R*[*F* ^2^ > 2σ(*F* ^2^)] = 0.045
*wR*(*F* ^2^) = 0.128
*S* = 1.05
2768 reflections
200 parameters
H-atom parameters constrained
Δρ_max_ = 0.51 e Å^−3^
Δρ_min_ = −0.27 e Å^−3^

### 

Data collection: *SMART* (Bruker, 2001 ▶); cell refinement: *SAINT* (Bruker, 2001 ▶); data reduction: *SAINT*; program(s) used to solve structure: *SHELXS97* (Sheldrick, 2008 ▶); program(s) used to refine structure: *SHELXL97* (Sheldrick, 2008 ▶); molecular graphics: *ORTEP-3* (Farrugia, 1997 ▶); software used to prepare material for publication: *SHELXL97*.

## Supplementary Material

Crystal structure: contains datablock(s) I, global. DOI: 10.1107/S1600536812017953/hb6751sup1.cif

Structure factors: contains datablock(s) I. DOI: 10.1107/S1600536812017953/hb6751Isup2.hkl

Supplementary material file. DOI: 10.1107/S1600536812017953/hb6751Isup3.cml

Additional supplementary materials:  crystallographic information; 3D view; checkCIF report

## Figures and Tables

**Table 1:** Hydrogen-bond geometry (Å, °)

*D*—H⋯*A*	*D*—H	H⋯*A*	*D*⋯*A*	*D*—H⋯*A*
C7—H7*B*⋯O5^i^	0.96	2.55	3.396 (4)	147
C7—H7*C*⋯O4^ii^	0.96	2.57	3.356 (3)	139
C13—H13⋯O3^iii^	0.93	2.50	3.411 (3)	168
C17—H17*B*⋯S2	0.97	2.52	3.160 (3)	124

Symmetry codes: (i) 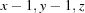; (ii) 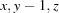; (iii) 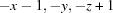.

**Fig. 1.**
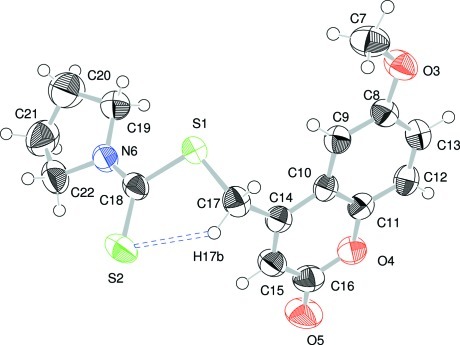
The molecular structure of the title compound. Displacement ellipsoids are drawn at the 50% probability level.

**Fig. 2.**
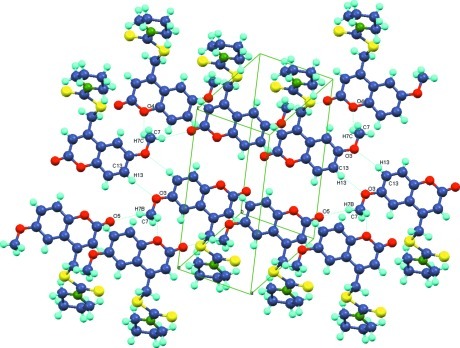
The packing of the molecules in the title structure.

## supplementary crystallographic information

### Comment

In continuation of our interest on crystal structures of coumarin derivatives (Kant *et al.*, 2012), we now report the crystal structure of the title compound.

The asymmetric unit of (6-methoxy-2-oxo-2H-chromen-4-yl)methyl pyrrolidine-1-carbodithioate is shown in Fig. 1. The 2H-chromene (O4/C8–C16) ring is close to planar, with a maximum deviation of 0.034 (2) Å for atom C16. The dihedral angle between the 2H-chromene (O4/C8–C16) ring and pyrrolidine (N6/C18–C22) ring is 75.24 (16)°.

In the crystal, (Fig. 2), the molecules are connected via weak C7—H7B···O5, C7—H7C···O4 and C13—H13···O3 interaction hydrogen bonds (Table 1). Furthermore, the crystal structure packing also exhibits π-π interactions, with distance of 3.6106 (13)Å between the centroids Cg3 (C8–C13) of the benzene rings of neighbouring molecules

### Experimental

4-bromomethyl coumarin required was synthesized according to an already reported procedure involving Pechmann cyclization of phenols with 4-Bromoethyl acetoacetate and sodium pyrrolidine-1-carbodithioate was synthesized according to the procedure reported. A mixture of 6-methoxy-4-bromomethyl coumarin (0.01 mol) and sodium pyrrolidine-1-carbodithioate (0.01 mol) in 30 ml dry alcohol was stirred for 24 hrs at room temperature (the reaction was monitored by TLC). The solvent was evaporated and the solid obtained was extracted twice with MDC-H_2_O mixture. The organic layer dried over anhydrous CaCl_2_ and on evaporating the organic solvent the title compound can be obtained. The compound was recrystallised from an ethanol-chloroform solvent mixture as colourless plates. Yield = 81%, M.P.435 K.

IR (KBr) 660 cm^-1^ (C—S), 1251 cm^-1^ (C=S), 1036 cm^-^1(C—O), 842 cm^-^1 (C—N),1279 cm^-1^ (C—O—C), 1708.6 cm^-1^ (C=O). GCMS: m/e: 335. 1H NMR (400 MHz, DMSO.D6, δ, p.p.m.): 1.92 (m,2*H*, C_10_), 2.01 ((m,2*H*, C_1_), 2.49(m,4*H*, C_2_,*C*_11_), 3.80 (s,3*H*, C_9_), 4.86 (s,2*H*, C_4_), 6.57 (s,1*H*, C_12_), 7.24(m,1*H*, C_15_), 7.36 (t,1*H*, C_7_), 7.38 (s,1*H*, C_16_). Elemental analysis: C, 57.26; H, 5.07; N, 4.15; O, 14.29; S, 19.08.

### Refinement

All H atoms were positioned at calculated positions C—H = 0.93 Å for aromatic H, C—H = 0.97 Å for methelene H and C—H = 0.96 Å for methyl H and refined using a riding model with *U*_iso_(H) = 1.5*U*_eq_(C)for methyl H and *U*_iso_(H) = 1.2*U*_eq_(C)for other H.

### Crystal data

**Table:**

C_16_H_17_NO_3_S_2_	*Z* = 2
*M**_r_* = 335.43	*F*(000) = 352
Triclinic, *P*1	*D*_x_ = 1.410 Mg m^−^^3^
Hall symbol: -P 1	Melting point: 435 K
*a* = 6.7223 (2) Å	Mo *K*α radiation, λ = 0.71073 Å
*b* = 8.0369 (2) Å	Cell parameters from 2768 reflections
*c* = 15.4101 (5) Å	θ = 2.7–25.0°
α = 75.320 (2)°	µ = 0.35 mm^−^^1^
β = 88.482 (1)°	*T* = 293 K
γ = 78.842 (1)°	Plate, colourless
*V* = 789.93 (4) Å^3^	0.24 × 0.20 × 0.12 mm

### Data collection

**Table:**

Bruker SMART CCD diffractometer	2768 independent reflections
Radiation source: fine-focus sealed tube	2453 reflections with *I* > 2σ(*I*)
Graphite monochromator	*R*_int_ = 0.024
ω and φ scans	θ_max_ = 25.0°, θ_min_ = 2.7°
Absorption correction: multi-scan (*SADABS*; Bruker, 2001)	*h* = −7→7
*T*_min_ = 0.770, *T*_max_ = 1.000	*k* = −9→9
15231 measured reflections	*l* = −18→18

### Refinement

**Table:**

Refinement on *F*^2^	Primary atom site location: structure-invariant direct methods
Least-squares matrix: full	Secondary atom site location: difference Fourier map
*R*[*F*^2^ > 2σ(*F*^2^)] = 0.045	Hydrogen site location: inferred from neighbouring sites
*wR*(*F*^2^) = 0.128	H-atom parameters constrained
*S* = 1.05	*w* = 1/[σ^2^(*F*_o_^2^) + (0.0674*P*)^2^ + 0.3732*P*] where *P* = (*F*_o_^2^ + 2*F*_c_^2^)/3
2768 reflections	(Δ/σ)_max_ < 0.001
200 parameters	Δρ_max_ = 0.51 e Å^−^^3^
0 restraints	Δρ_min_ = −0.27 e Å^−^^3^

### Special details

**Table:**

Geometry. All s.u.'s (except the s.u. in the dihedral angle between two l.s. planes) are estimated using the full covariance matrix. The cell s.u.'s are taken into account individually in the estimation of s.u.'s in distances, angles and torsion angles; correlations between s.u.'s in cell parameters are only used when they are defined by crystal symmetry. An approximate (isotropic) treatment of cell s.u.'s is used for estimating s.u.'s involving l.s. planes.
Refinement. Refinement of *F*^2^ against ALL reflections. The weighted *R*-factor *wR* and goodness of fit *S* are based on *F*^2^, conventional *R*-factors *R* are based on *F*, with *F* set to zero for negative *F*^2^. The threshold expression of *F*^2^ > 2σ(*F*^2^) is used only for calculating *R*-factors(gt) *etc*. and is not relevant to the choice of reflections for refinement. *R*-factors based on *F*^2^ are statistically about twice as large as those based on *F*, and *R*- factors based on ALL data will be even larger.

### Fractional atomic coordinates and isotropic or equivalent isotropic displacement parameters (Å^2^)

**Table:**

	*x*	*y*	*z*	*U*_iso_*/*U*_eq_	
S1	0.19010 (9)	0.10311 (9)	0.13745 (4)	0.0584 (2)	
S2	0.60578 (10)	0.18643 (10)	0.08087 (5)	0.0710 (2)	
O3	−0.3276 (3)	−0.0847 (2)	0.41586 (13)	0.0658 (5)	
O4	0.1152 (3)	0.4294 (2)	0.39350 (11)	0.0574 (4)	
O5	0.3545 (4)	0.5741 (3)	0.34066 (16)	0.0881 (6)	
N6	0.2772 (3)	0.2620 (2)	−0.02227 (13)	0.0522 (5)	
C7	−0.2661 (5)	−0.2251 (3)	0.3765 (2)	0.0716 (7)	
H7A	−0.2656	−0.1814	0.3124	0.107*	
H7B	−0.3585	−0.3048	0.3919	0.107*	
H7C	−0.1321	−0.2852	0.3980	0.107*	
C8	−0.2095 (3)	0.0380 (3)	0.40578 (15)	0.0495 (5)	
C9	−0.0408 (3)	0.0425 (3)	0.35217 (14)	0.0471 (5)	
H9	−0.0035	−0.0412	0.3197	0.057*	
C10	0.0731 (3)	0.1724 (3)	0.34692 (13)	0.0434 (5)	
C11	0.0111 (4)	0.2970 (3)	0.39567 (14)	0.0473 (5)	
C12	−0.1572 (4)	0.2920 (3)	0.44935 (15)	0.0540 (6)	
H12	−0.1956	0.3754	0.4820	0.065*	
C13	−0.2659 (4)	0.1638 (3)	0.45391 (15)	0.0545 (6)	
H13	−0.3794	0.1603	0.4897	0.065*	
C14	0.2545 (3)	0.1843 (3)	0.29518 (14)	0.0471 (5)	
C15	0.3517 (4)	0.3155 (3)	0.29444 (16)	0.0572 (6)	
H15	0.4696	0.3210	0.2618	0.069*	
C16	0.2814 (4)	0.4486 (3)	0.34202 (17)	0.0610 (6)	
C17	0.3306 (4)	0.0515 (3)	0.24279 (15)	0.0546 (6)	
H17A	0.3167	−0.0640	0.2780	0.066*	
H17B	0.4734	0.0496	0.2309	0.066*	
C18	0.3634 (3)	0.1932 (3)	0.05759 (16)	0.0493 (5)	
C19	0.0669 (4)	0.2655 (4)	−0.04648 (18)	0.0676 (7)	
H19A	−0.0266	0.3443	−0.0191	0.081*	
H19B	0.0347	0.1493	−0.0275	0.081*	
C20	0.0557 (6)	0.3305 (6)	−0.1473 (2)	0.1022 (12)	
H20A	0.0747	0.2328	−0.1749	0.123*	
H20B	−0.0747	0.4054	−0.1670	0.123*	
C21	0.2206 (6)	0.4298 (5)	−0.1712 (2)	0.0920 (10)	
H21A	0.1743	0.5511	−0.1698	0.110*	
H21B	0.2682	0.4266	−0.2309	0.110*	
C22	0.3867 (5)	0.3409 (4)	−0.10192 (18)	0.0695 (7)	
H22A	0.4816	0.2516	−0.1219	0.083*	
H22B	0.4600	0.4251	−0.0891	0.083*	

### Atomic displacement parameters (Å^2^)

**Table:**

	*U*^11^	*U*^22^	*U*^33^	*U*^12^	*U*^13^	*U*^23^
S1	0.0516 (4)	0.0805 (4)	0.0513 (3)	−0.0254 (3)	0.0134 (3)	−0.0233 (3)
S2	0.0432 (4)	0.0889 (5)	0.0828 (5)	−0.0197 (3)	0.0108 (3)	−0.0209 (4)
O3	0.0625 (11)	0.0579 (10)	0.0844 (12)	−0.0230 (8)	0.0155 (9)	−0.0240 (9)
O4	0.0653 (11)	0.0497 (9)	0.0624 (10)	−0.0165 (8)	0.0038 (8)	−0.0199 (7)
O5	0.0919 (15)	0.0733 (12)	0.1171 (17)	−0.0450 (11)	0.0114 (13)	−0.0359 (12)
N6	0.0517 (11)	0.0552 (11)	0.0534 (11)	−0.0160 (8)	0.0119 (8)	−0.0175 (9)
C7	0.0665 (17)	0.0551 (14)	0.099 (2)	−0.0158 (12)	−0.0006 (15)	−0.0272 (14)
C8	0.0487 (13)	0.0462 (11)	0.0509 (12)	−0.0090 (9)	0.0005 (10)	−0.0075 (9)
C9	0.0518 (13)	0.0424 (11)	0.0473 (11)	−0.0075 (9)	0.0025 (9)	−0.0132 (9)
C10	0.0484 (12)	0.0408 (10)	0.0369 (10)	−0.0040 (9)	−0.0026 (8)	−0.0054 (8)
C11	0.0553 (13)	0.0415 (11)	0.0442 (11)	−0.0092 (9)	−0.0033 (9)	−0.0090 (9)
C12	0.0631 (15)	0.0511 (12)	0.0474 (12)	−0.0040 (11)	0.0057 (10)	−0.0177 (10)
C13	0.0551 (14)	0.0547 (13)	0.0515 (12)	−0.0088 (11)	0.0102 (10)	−0.0121 (10)
C14	0.0474 (12)	0.0462 (11)	0.0444 (11)	−0.0082 (9)	−0.0024 (9)	−0.0060 (9)
C15	0.0522 (14)	0.0604 (14)	0.0595 (14)	−0.0175 (11)	0.0047 (11)	−0.0117 (11)
C16	0.0641 (16)	0.0542 (13)	0.0669 (15)	−0.0193 (12)	−0.0062 (12)	−0.0128 (11)
C17	0.0499 (13)	0.0567 (13)	0.0545 (13)	−0.0064 (10)	0.0067 (10)	−0.0124 (10)
C18	0.0462 (13)	0.0469 (11)	0.0611 (13)	−0.0122 (9)	0.0137 (10)	−0.0237 (10)
C19	0.0595 (16)	0.0845 (18)	0.0611 (15)	−0.0181 (13)	0.0014 (12)	−0.0194 (13)
C20	0.105 (3)	0.130 (3)	0.0654 (19)	−0.038 (2)	−0.0115 (18)	0.0000 (19)
C21	0.120 (3)	0.095 (2)	0.0615 (17)	−0.038 (2)	0.0020 (18)	−0.0095 (16)
C22	0.0791 (19)	0.0671 (15)	0.0659 (16)	−0.0263 (14)	0.0254 (14)	−0.0168 (13)

### Geometric parameters (Å, º)

**Table:**

S1—C18	1.787 (2)	C12—C13	1.361 (3)
S1—C17	1.813 (2)	C12—H12	0.9300
S2—C18	1.666 (2)	C13—H13	0.9300
O3—C8	1.358 (3)	C14—C15	1.341 (3)
O3—C7	1.403 (3)	C14—C17	1.502 (3)
O4—C16	1.364 (3)	C15—C16	1.446 (4)
O4—C11	1.375 (3)	C15—H15	0.9300
O5—C16	1.199 (3)	C17—H17A	0.9700
N6—C18	1.313 (3)	C17—H17B	0.9700
N6—C19	1.465 (3)	C19—C20	1.507 (4)
N6—C22	1.480 (3)	C19—H19A	0.9700
C7—H7A	0.9600	C19—H19B	0.9700
C7—H7B	0.9600	C20—C21	1.474 (5)
C7—H7C	0.9600	C20—H20A	0.9700
C8—C9	1.386 (3)	C20—H20B	0.9700
C8—C13	1.391 (3)	C21—C22	1.505 (5)
C9—C10	1.395 (3)	C21—H21A	0.9700
C9—H9	0.9300	C21—H21B	0.9700
C10—C11	1.395 (3)	C22—H22A	0.9700
C10—C14	1.445 (3)	C22—H22B	0.9700
C11—C12	1.385 (3)		
			
C18—S1—C17	102.70 (11)	O5—C16—O4	116.9 (2)
C8—O3—C7	118.4 (2)	O5—C16—C15	126.6 (3)
C16—O4—C11	121.98 (18)	O4—C16—C15	116.5 (2)
C18—N6—C19	126.0 (2)	C14—C17—S1	110.94 (16)
C18—N6—C22	123.3 (2)	C14—C17—H17A	109.5
C19—N6—C22	110.6 (2)	S1—C17—H17A	109.5
O3—C7—H7A	109.5	C14—C17—H17B	109.5
O3—C7—H7B	109.5	S1—C17—H17B	109.5
H7A—C7—H7B	109.5	H17A—C17—H17B	108.0
O3—C7—H7C	109.5	N6—C18—S2	124.10 (18)
H7A—C7—H7C	109.5	N6—C18—S1	111.67 (17)
H7B—C7—H7C	109.5	S2—C18—S1	124.21 (15)
O3—C8—C9	124.1 (2)	N6—C19—C20	104.6 (2)
O3—C8—C13	115.9 (2)	N6—C19—H19A	110.8
C9—C8—C13	119.9 (2)	C20—C19—H19A	110.8
C8—C9—C10	120.1 (2)	N6—C19—H19B	110.8
C8—C9—H9	120.0	C20—C19—H19B	110.8
C10—C9—H9	120.0	H19A—C19—H19B	108.9
C11—C10—C9	118.4 (2)	C21—C20—C19	105.7 (3)
C11—C10—C14	117.6 (2)	C21—C20—H20A	110.6
C9—C10—C14	123.99 (19)	C19—C20—H20A	110.6
O4—C11—C12	116.88 (19)	C21—C20—H20B	110.6
O4—C11—C10	121.7 (2)	C19—C20—H20B	110.6
C12—C11—C10	121.4 (2)	H20A—C20—H20B	108.7
C13—C12—C11	119.3 (2)	C20—C21—C22	105.6 (3)
C13—C12—H12	120.3	C20—C21—H21A	110.6
C11—C12—H12	120.3	C22—C21—H21A	110.6
C12—C13—C8	120.9 (2)	C20—C21—H21B	110.6
C12—C13—H13	119.6	C22—C21—H21B	110.6
C8—C13—H13	119.6	H21A—C21—H21B	108.8
C15—C14—C10	119.0 (2)	N6—C22—C21	103.7 (2)
C15—C14—C17	121.1 (2)	N6—C22—H22A	111.0
C10—C14—C17	119.93 (19)	C21—C22—H22A	111.0
C14—C15—C16	123.0 (2)	N6—C22—H22B	111.0
C14—C15—H15	118.5	C21—C22—H22B	111.0
C16—C15—H15	118.5	H22A—C22—H22B	109.0

### Hydrogen-bond geometry (Å, º)

**Table:**

*D*—H···*A*	*D*—H	H···*A*	*D*···*A*	*D*—H···*A*
C7—H7*B*···O5^i^	0.96	2.55	3.396 (4)	147
C7—H7*C*···O4^ii^	0.96	2.57	3.356 (3)	139
C13—H13···O3^iii^	0.93	2.50	3.411 (3)	168
C17—H17*B*···S2	0.97	2.52	3.160 (3)	124

Symmetry codes: (i) *x*−1, *y*−1, *z*; (ii) *x*, *y*−1, *z*; (iii) −*x*−1, −*y*, −*z*+1.
